# Pioneering adsorption-assisted electrochemical polymerization approach for the fabrication of an acetaminophen sensor: experimental and theoretical studies

**DOI:** 10.1039/d5ra07827j

**Published:** 2025-12-11

**Authors:** Saheed E. Elugoke, Usisipho Feleni, Abolanle S. Adekunle, Thabo T. I. Nkambule, Bhekie B. Mamba, Eno E. Ebenso

**Affiliations:** a Centre for Materials Science, College of Science, Engineering and Technology (CSET) Johannesburg 1710 South Africa elugokesaheed@gmail.com elugose@unisa.ac.za ebensee@unisa.ac.za; b Institute for Nanotechnology and Water Sustainability, College of Science, Engineering and Technology (CSET) Johannesburg 1710 South Africa; c Institute for Catalysis and Energy Solutions (ICES), College of Science, Engineering and Technology Johannesburg 1710 South Africa

## Abstract

The fabrication of a polymelamine (PM)-modified glassy carbon electrode (GCE) *via* an innovative adsorption-assisted electropolymerization approach (EPM/GCE) for the detection of acetaminophen is presented in this study. Electrooxidation of ACE at EPM/GCE and another PM-modified GCE produced through the conventional electrodeposition approach (CPM/GCE) showed that the EPM/GCE possessed superior electrocatalytic activity toward ACE oxidation. Investigation of the impact of ACE concentration on the peak current showed that ACE concentration was linear over the range of 1.58–7.86 and 15.57–158.8 µM. The limit of detection (LOD) for ACE at the EPM/GCE was estimated from the lower linear range as 1.46 µM. ACE electroanalysis in a drug sample showed a progressive increase in ACE concentration with drug dosage, while the percentage recovery of ACE from the wastewater sample was 92%. The increase in the peak current of ACE with an increase in ACE concentration in the presence of ciprofloxacin (CFX) showed that the proposed sensor is capable of ACE determination in the presence of another drug sample. Simulation of the adsorption of ACE and CFX on the proposed sensor (represented by melamine) revealed that it has a greater affinity for CFX than ACE, suggesting its suitability for CFX determination in addition to ACE. Density functional theory (DFT) calculations also confirmed the suitability of melamine for GCE modification. Adsorption simulations revealed that the affinity of the proposed sensor for CFX is due to the higher reactivity of CFX than that of ACE. EPM/GCE showed remarkable repeatability, reproducibility and stability, confirming its great potential for routine drug analysis in real-life samples.

## Abstract 2

Electrochemical detection of acetaminophen using at a glassy carbon electrode (GCE) modified with polymelamine *via* an adsorption-assisted electropolymerization approach was attempted.

## Graphical abstract

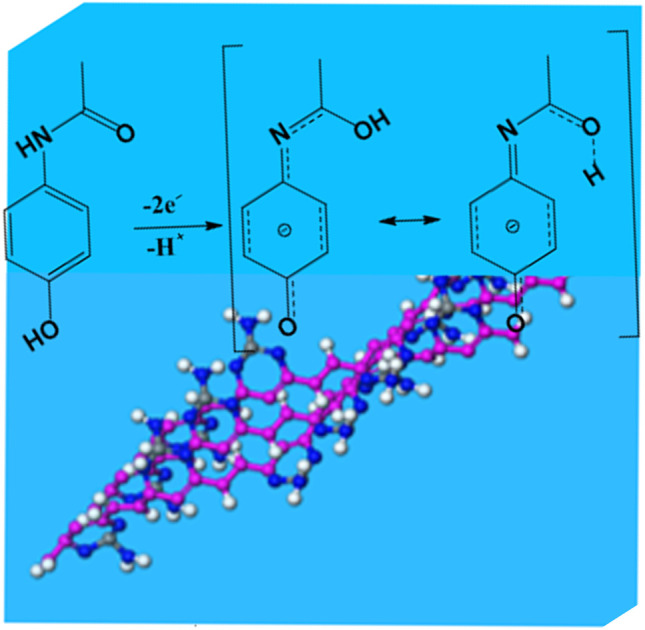

## 1. Introduction

Acetaminophen (ACE), an organic compound commonly referred to as paracetamol, is the most popular pain-relieving over-the-counter (OTC) drug. In addition to being an OTC drug, ACE-containing drugs are cheap and easily accessible, making their abuse a global phenomenon. Similar to other pain killers, the excessive use of ACE over time can lead to chronic ailments such as kidney damage and liver malfunction and may lead to other life-threatening illnesses.^1,2^ Besides deliberate ACE abuse, ACE quality assurance at the point of production may also be instrumental to curtailing the risks of human exposure to high ACE dosages. It is also worth noting that inappropriate clinical and industrial waste disposal are anthropogenic activities that are actively increasing the ACE load in waterbodies.^3^ Therefore, the development of devices for monitoring the ACE content in paracetamol brands and the quantity of ACE in water samples is a necessity.

Attempts at determining the ACE content in real-life samples have been made using different analytical methods including chromatography,^4^ fluorimetry,^5^ electroanalytical^6^ and electrochemiluminescence^7^ methods. Among them, the advantages of the electroanalytical method, such as its high sensitivity, simplicity, and cost-effectiveness, have made it the preferred analytical method for ACE determination in the last few years.^6^ Notably, electrochemical sensors fabricated with metal oxides, polymers, carbon nanomaterials, spinel ferrites and MXenes are sensitive and efficient electroanalytical devices, which have been previously deployed for ACE determination in real-life samples.^8–12^ These electrochemical sensors were fabricated using various techniques including drop-casting, bulk modification and electrodeposition. The electrodeposition approach offers reusability and limited physical interference by researchers, among other advantages over the popular drop-casting method. Consequently, a wide range of electrochemical sensors with exceptional stability and low limit of detection (LOD) has been developed following the electrodeposition protocol.^12–17^ The conventional electrodeposition method involves the electrochemical deposition of a polymer film from a solution containing the monomer and the electrolyte or a solvent, as shown in the electrodeposition of polymer films from several dye and amino acid molecules in solution.^12–20^ Herein, the adsorption-assisted electropolymerization of melamine using a pristine electrode pre-modified with the monomer molecules is presented. In addition to the extremely small amount of monomer required for this modification technique, it has the tendency to produce highly stable polymer films. Melamine was adopted as the monomer molecule because of the identity of polymelamine as a conducting polymer^21,22^ and the possibility of achieving signal amplification *via* the π–π interaction between the melamine ring and the aromatic component of ACE. To the best of our knowledge, the findings herein represent the pioneering adsorption-assisted electrochemical polymerization of melamine and the adoption of the resultant electroanalytical device for analysing ACE in a paracetamol brand and wastewater sample.

## 2. Experimental

### 2.1 Materials

Analytical grade melamine (97%), acetaminophen (≥99%), sodium dihydrogen phosphate (≥98% Na_2_HPO_4_), disodium hydrogen phosphate (≥98% NaH_2_PO_4_), hydrochloric acid (37% HCl), sodium hydroxide (≥99%), and ciprofloxacin (≥98% CFX). Electrochemical experiments were conducted using a PalmSens potentiostat with the PSTrace software. The three electrodes were connected to the potentiostat including a saturated calomel reference electrode, platinum counter electrode and modified glassy carbon working electrode (GCE, diameter = 2 mm). A PerkinElmer Frontier Fourier-transform infrared spectrophotometer was used to record the Fourier transform infrared (FT-IR) spectra of the monomer. Scanning electron microscopy (SEM) was performed using a JEOL JSM microscope at an acceleration voltage of 15 kV. All electrochemical experiments were carried out in phosphate buffer, unless otherwise stated.

### 2.2 Preparation of the drug sample

A paracetamol capsule manufactured by Adcock Ingram (South Africa) was used for the real sample analysis in this study. The powder in the capsule was extracted and measured to confirm the quoted weight on the drug's packaging material. This powder weighed about 617 mg, out of which 500 mg was ACE and the remaining 117 mg were excipients such as potassium sorbate and magnesium stearate. As stated on the pack of the tablet, the capsule contained about 81% ACE, which is in agreement with our measurement. An appropriate amount of the drug sample was dissolved in deionized water to prepare the desired concentration of the drug. Notably, the entire drug sample dissolved in deionized water without any visible residue. Except when water samples were spiked with the drug, the drug sample was directly used for ACE electroanalysis.

### 2.3 Theoretical studies

The background for the application of quantum chemical parameters obtained from density functional theory (DFT) in the determination of the molecular properties of the monomer molecules was presented in our previous publication on ACE determination.^23^ The electronegativity (*χ*), ionization energy (IE), electron-donating power (*ω*^−^), hardness (*η*), electron-accepting power (*ω*^+^), electron affinity (EA), softness (*σ*) and chemical potential (*µ*) of melamine were obtained from the highest occupied molecular orbital (HOMO) and the lowest unoccupied molecular orbital (LUMO) energies of melamine, as illustrated in eqn (1)–(8).

1 EA *=* −*E*_LUMO_

2 IE*=* −*E*_HOMO_

3 *η =* (IE – EA)/2

4 *σ =* 1/*η*

5 *µ* = −[(IE + EA)/2]

6 *χ =* −*µ*

7 *ω*^+^*=* (IE + 3EA)^2^/(16(IE − EA))

8 *ω*^−^*=* (3IE + EA)^2^/(16(IE − EA))

The HOMO and LUMO energies and the Fukui indices for nucleophilic attack (*f*_k_^+^), electrophilic attack (*f*_k_^−^) and radical attack (*f*^0^_k_) were calculated using the Dmol3 module in the Material Studio software. These calculations and geometry optimization of all the structures in this study were done using the DND basis set with the generalized gradient approximation (GGA) method for executing the dependence of the Becke (exchange)–Lee, Yang and Parr (correlation) functional on electron density gradient (GGA/BLYP). Adsorption studies were conducted with the adsorption locator module using the appropriate adsorbate, while the melamine molecule represents the adsorbent.

## 3. Results and discussion

### 3.1 Computational studies

**Fig. 1:**
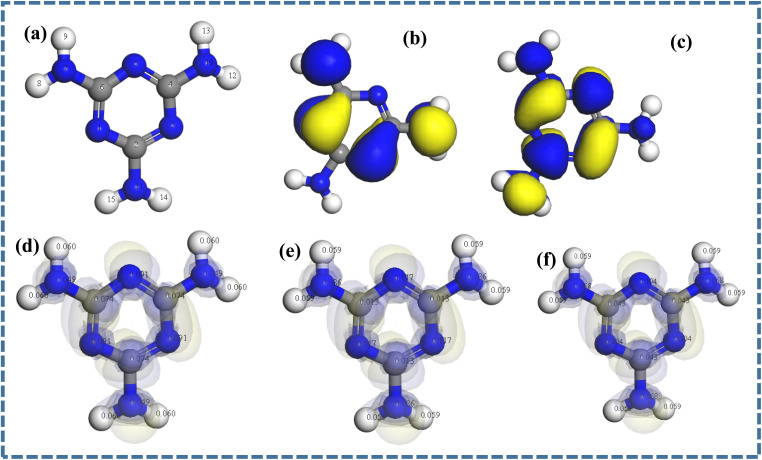
(a) Optimized structure and (b and c) frontier molecular orbitals of melamine. Optimized melamine structure with Fukui indices: (d) *f*_k_^+^, (e) *f*_k_^−^ and (f) *f*^0^_k_.

Density functional theory (DFT) calculations were performed to identify the active sites on melamine and its reactivity at the molecular level. Fig. 1a–c show the optimized structure of melamine with the server code and the respective HOMO and LUMO. The frontier molecular orbitals of melamine (HOMO and LUMO) obtained after geometry optimization of the monomer revealed that the HOMO and the LUMO were localized at the triazine ring and the nitrogen atoms of the amino groups (Fig. 1b and c), respectively. Compared to the HOMO–LUMO energy gap (Δ*E*) of other monomers such as benzoguanamine (8.122 eV),^23^ a lower energy gap was obtained for melamine (4.954), suggesting that melamine is more reactive, and could easily undergo electropolymerization. The small Δ*E* of melamine also indicates that little energy will be needed for the transfer of an electron from its HOMO to the LUMO of the amorphous carbon that makes up the glassy carbon electrode (GCE). Interestingly, the calculated electron affinity of melamine was extremely low (0.407), while its *χ* is relatively high, confirming its reluctance to accept an electron.

The harness (*η*) and softness (*σ*) values indicate the extent of the reactivity of a material. A low *η* value and a high *σ* value are expected of a very reactive compound.^23^ The corresponding values of *η* and *σ* were estimated to be 2.477 and 0.404, respectively, confirming the good reactivity and remarkable affinity of melamine for the bare GCE. Noteworthy, the value of *σ* recorded for melamine is higher than that of thymol blue, pyrogallol and benzoguanamine^23–25^ used for the modification of carbon paste electrode. The *ω*^+^ and *ω*^−^ of a compound gives insight into its extent of electron loss or gain. A compound with a superior *ω*^−^ is largely an electron-donating entity. As shown in Table 1, the *ω*^−^ of melamine is higher than its *ω*^+^, confirming the electron-donating ability of this monomer.

**Table 1:** Quantum chemical parameters of melamine

Parameter	Value
*E*_HOMO_ (eV)	−5.361
*E*_LUMO_ (eV)	−0.407
Δ*E*	4.954
Electron affinity (EA)	0.407
Electronegativity (*χ*)	2.884
Softness (*σ*)	0.404
Hardness (*η*)	2.477
Chemical potential (*µ*)	−2.884
Ionization energy (IE)	5.361
Electron donating power (*ω*^−^)	3.431
Electron accepting power (*ω*^+^)	0.547

The Fukui indices of melamine were calculated to identify the active sites on the monomer. As shown in Fig. 1d, higher *f*_k_^+^ values were recorded within the triazine ring than other parts of the monomer, suggesting that the preferred site for nucleophilic attacks are the C and N atoms of the triazine ring. Meanwhile, the nitrogen atoms of the triazine ring and those of the amino groups are the preferred sites for electrophilic and radical attack (Fig. 1e and f), respectively. These outcomes are indications that all the nitrogen atoms on melamine are active sites for electrophilic attack, confirming that they are available for interaction with the surface of the GCE. Also, considering the strong electron-donating tendencies of melamine, the triazine ring may be the preferable site for bond formation with the bare GCE and a viable site for bond formation with electrophilic compounds.

### 3.2 Microscopic and spectroscopic characterization

**Fig. 2:**
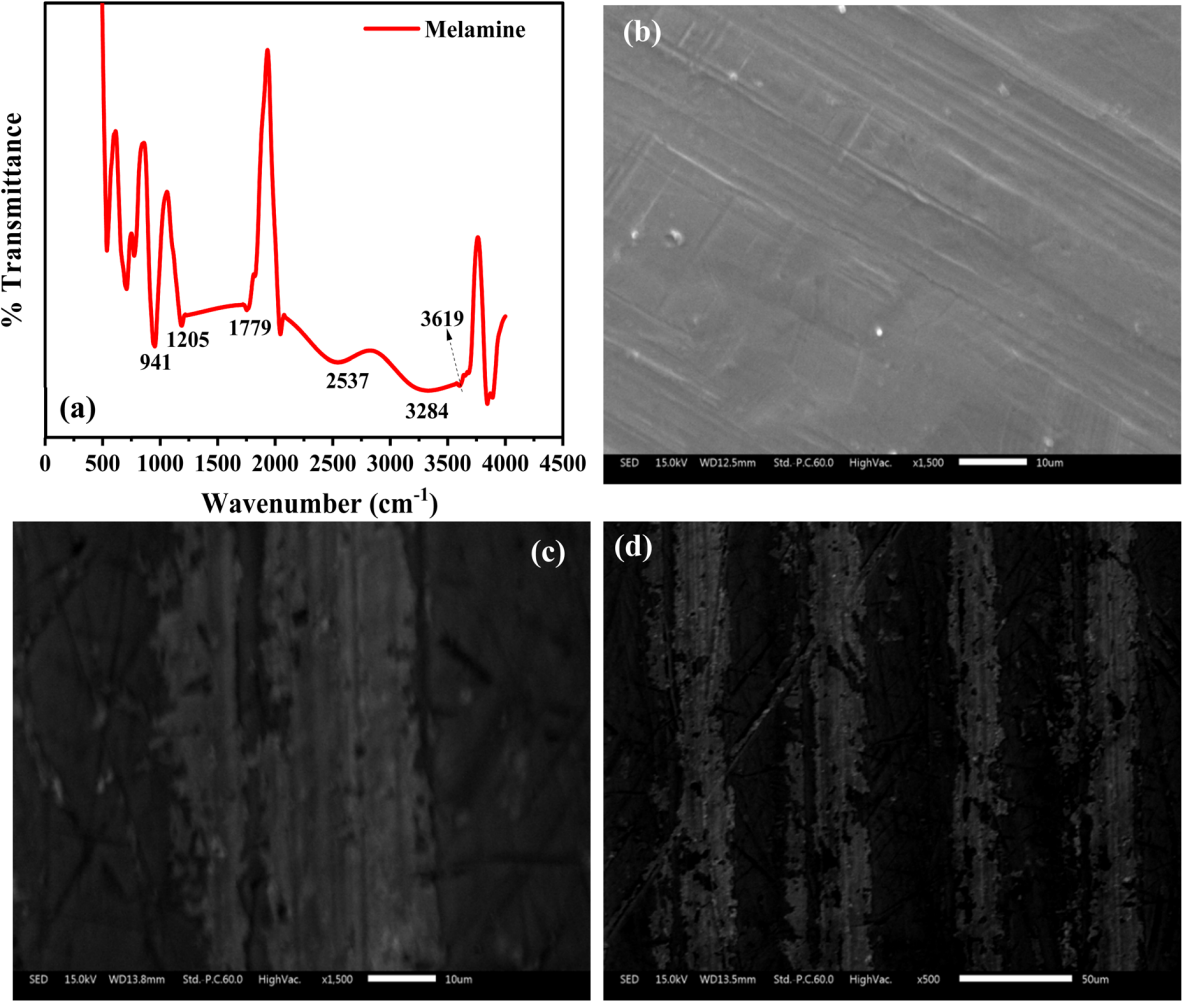
(a) FT-IR spectrum of melamine. SEM images of the bare electrode (b) and modified electrodes (c and d) at different magnifications.

FT-IR analysis of melamine was done to confirm the identity of the monomer used for the polymerization process. Fig. 2a revealed absorption peaks located at 941, 1205, 1779, 2537, 3284 and 3619 cm^−1^ in the spectrum of melamine. The peaks at 3284 and 3619 cm^−1^ are due to the N–H stretch, while the peak at 1205 cm^−1^ is due to the C–N bond. The sharp absorption peak at 1779 cm^−1^ can be ascribed to the C=N bond, confirming the presence of a triazine ring in melamine. Also, the deformation peak of the triazine ring emerged at 820 cm^−1^, confirming that the monomer used in this study was melamine.

The SEM characterization of the bare electrode and the modified electrode is presented in Fig. 2b–d. The SEM image of the bare electrode revealed that it has a smoother morphology than the modified electrode (Fig. 2b), which has a micrograph that revealed the deposition of the polymer film (Fig. 2c). At a lower magnification, it was evident that the polymer films were deposited according to a regular pattern (Fig. 2d). These images confirmed the deposition of the polymer film on the surface of the bare electrode.

### 3.3 Conventional and adsorption-assisted electropolymerization of polymelamine

Electrochemical deposition of polymelamine (PM) was done using the conventional electrodeposition and the newly developed adsorption-assisted electropolymerization technique. Before the electropolymerization of melamine, the GCE was oxidized in phosphate buffer by applying a potential of +2.0 V for 300 s *via* chronoamperometry, as shown in Fig. S1a.^26^ GCE oxidation was necessary to enhance the adsorption of PM to the GCE. After GCE oxidation, the next step in the adsorption-assisted electropolymerization of PM was the adsorption of melamine onto the surface of the oxidized GCE by modifying the surface of the oxidized GCE with 1 mM melamine, and subsequently drying the modified GCE in an oven at 60 °C. Afterwards, the electrode was subjected to electropolymerization in 0.1 M HCl *via* cyclic voltammetry (CV) at 100 mV s^−1^ over 14 cycles (Fig. S1b). The resultant modified electrode was labelled EPM/GCE. Optimization of the number of cycles and the concentration of melamine required for the optimum performance of EPM/GCE was conducted by varying these parameters and applying the resultant electrode for the electroanalysis of 7.62 µM ACE. As shown in Fig. S1c and d, the highest peak current was recorded after 7 cycles but weak redox peaks were obtained. In particular, the reduction peak of ACE was barely visible when the EPM/GCE fabricated after 7 cycles was used. Consequently, the EPM/GCE fabricated with 14 CV cycles, which offered relatively sharper redox peaks and an anodic peak current close to that of the 7-cycle electrode, was selected. Further, using 14 CV cycles with varying melamine concentrations (1–4 mM) for the initial adsorption step, four different EPM/GCE were fabricated and applied for ACE electrooxidation. As shown in Fig. S1e and f, the best anodic peak current (*I*_pa_) was recorded with the sensor fabricated with 1 mM melamine. However, this sensor offered multiple reduction peaks, which may be due to the participation of more than one ACE redox pair in the electrooxidation process on the sensor. Consequently, the sensor fabricated with 2 mM melamine, which offered a comparable peak current and well-defined redox peaks, was selected for the further electroanalysis of ACE. Based on these outcomes, 2 mM melamine and 14 CV cycles were considered the optimum conditions for PM electrodeposition.

**Scheme 1:**
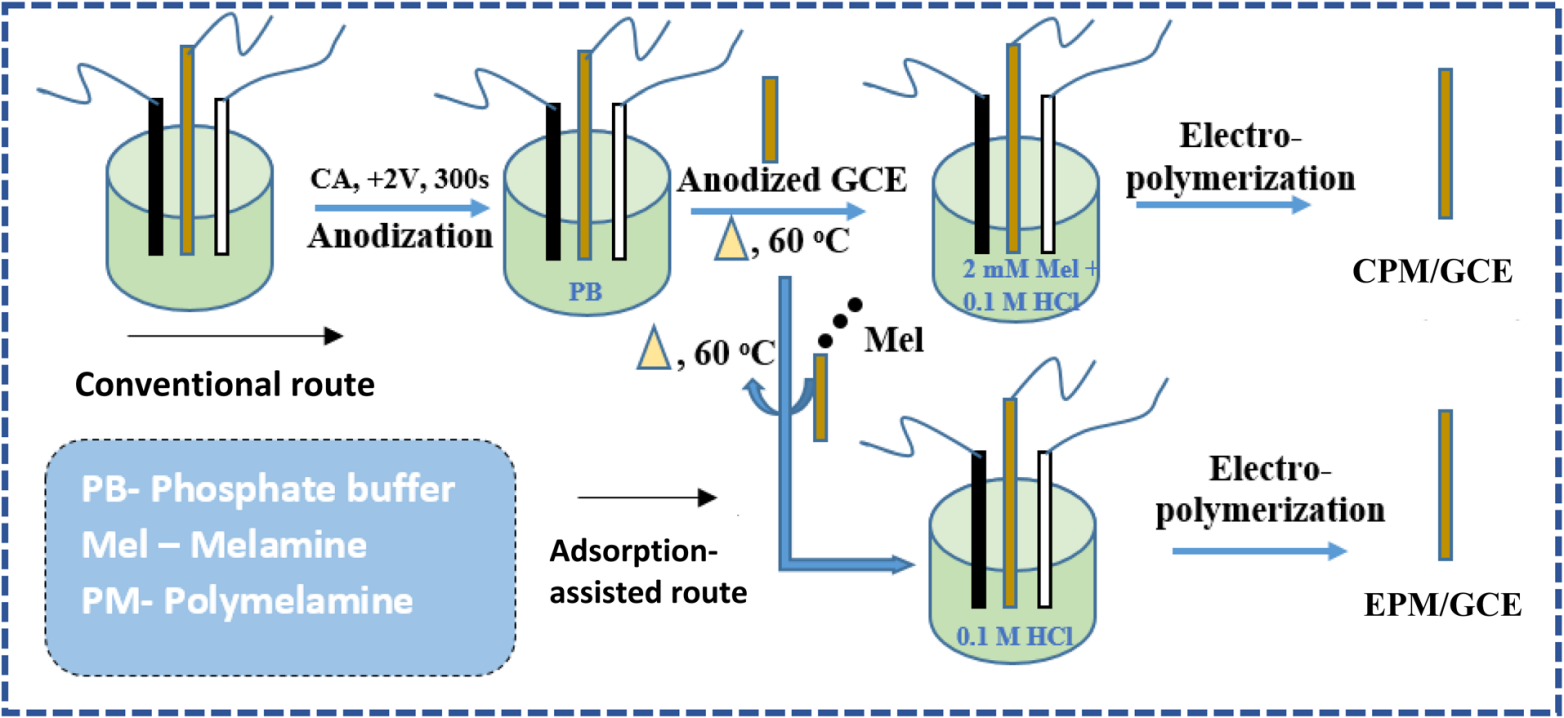
Fabrication of PM-modified electrode *via* the conventional and adsorption-assisted electropolymerization methods.

For comparison, the conventional electrodeposition approach was used for the electropolymerization of PM. An oxidized GCE was dried in a dry air oven and subjected to electropolymerization by cyclic voltammetry in the presence of 2 mM melamine at 100 mV s^−1^ for 14 cycles (Fig. S2). Similar to the fabrication of EPM/GCE, electropolymerization was performed in a cell containing 0.1 M HCl. The resultant PM-modified GCE labelled CPM/GCE was dried in an oven at 60 °C and stored at room temperature for electroanalysis. A schematic representation of the electrode modification processes is illustrated in Scheme 1.

**Scheme 2:**
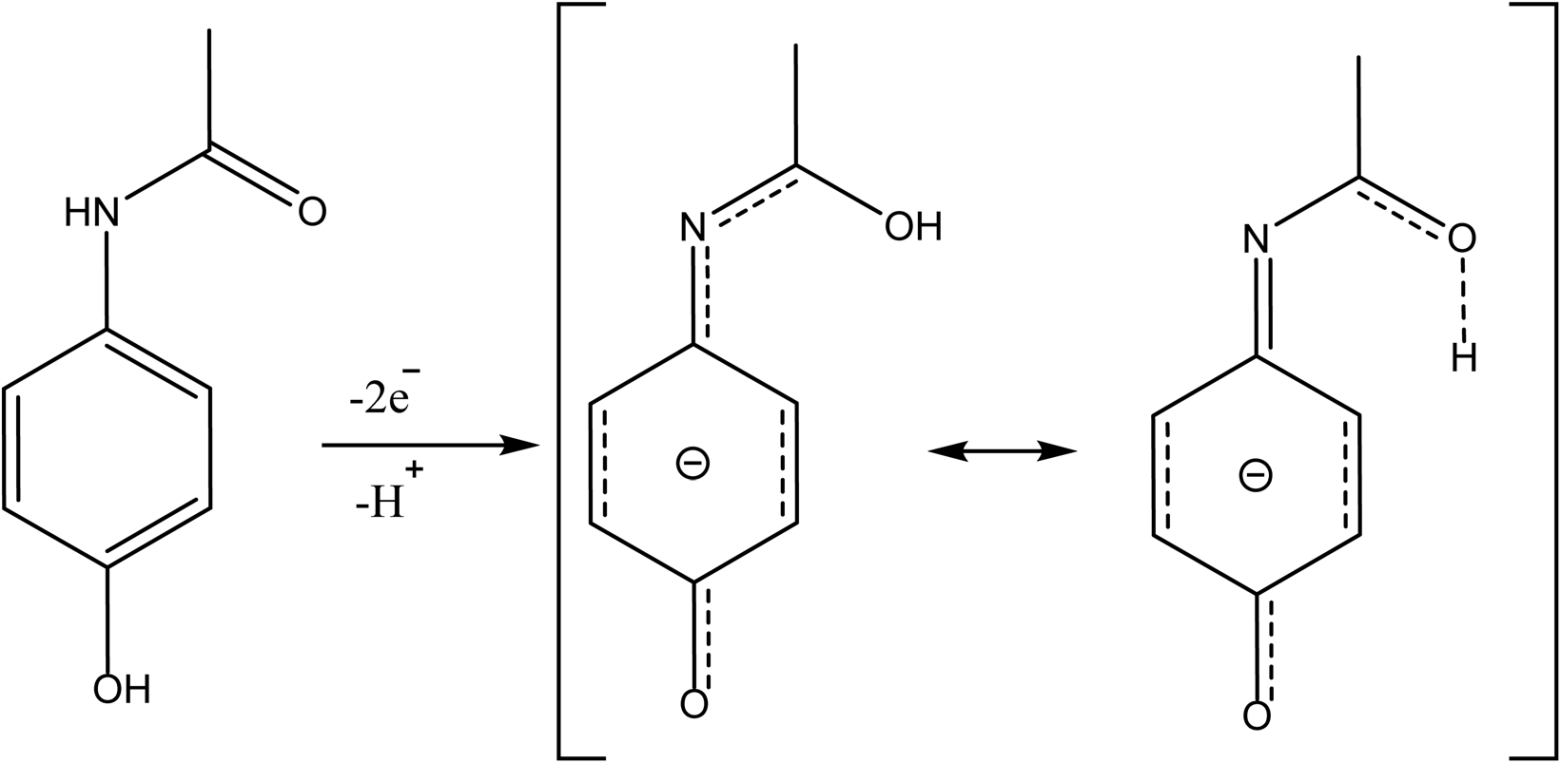
Mechanism of ACE oxidation.

**Fig. 3:**
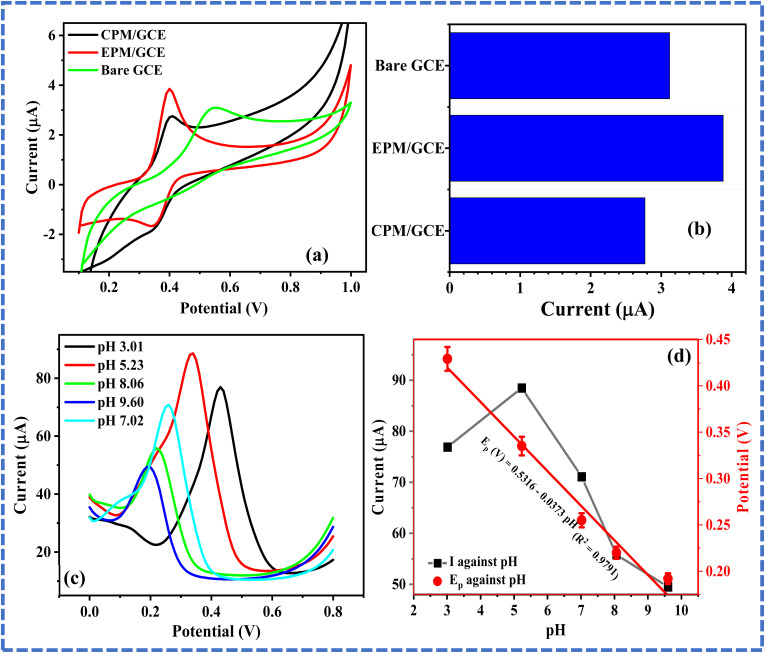
(a) Cyclic voltammograms of 0.16 mM ACE at bare GCE, CPM/GCE and EPM/GCE. (b) Chart showing the peak currents recorded from (a). (c) Differential pulse voltammogram of 15 µM ACE at EPM/GCE in phosphate buffer of varying pH (3.01–9.6) and (d) plots of ACE peak current and corresponding peak potentials against buffer pH.

ACE electrooxidation at the surface of the modified electrodes was used for a comparative study of the behavior of the electrodes in the presence of 0.16 mM ACE. Notably, ACE electroanalysis is usually a reversible process, as depicted in Scheme 2. As depicted in Fig. 3a and b, the stable anodic ACE peak current (*I*_pa_) obtained at the EPM/GCE was about 1.4- and 1.2-times higher than the *I*_pa_ at CPM and the bare GCE, respectively. This outcome suggests that the modification of the bare electrode with PM *via* the adsorption-assisted route improved the electrocatalytic activity of the electrode toward ACE oxidation. It is also worth noting that the bare GCE offered a lower cathodic peak current than the modified electrodes, suggesting that the modification of the bare GCE with PM improved the electrocatalytic reduction of ACE, irrespective of the modification method adopted. Moreover, the *I*_pa_ recorded at CPM and EPM was obtained at 0.39 V, while the *I*_pa_ at the bare GCE emerged at 0.52 V, suggesting that the modification of the electrode with PM enabled the detection of ACE with a lower overpotential, indicating improved electron transport at the modified electrodes. Particularly, the π–π interaction between PM and ACE contributed to the improved redox peaks and electron transport at the modified electrodes. The superiority of EPM/GCE over CPM/GCE can be attributed to presence of more active sites for ACE electroanalysis on EPM/GCE than that on CPM/GCE. Based on these outcomes, EPM/GCE was selected for subsequent ACE electroanalysis.

### 3.4 pH optimization

The pH of the phosphate buffer electrolyte was optimized by carrying out the electroanalysis of ACE in varying buffer pH (pH 3.01–9.6) using differential pulse voltammetry (DPV). As shown in Fig. 3c, the peak current of ACE changed with a variation in the pH of the electrolyte. Fig. 3d revealed that the peak current increased with an increase in pH from 3.01–5.23 and consistently dropped in intensity over a pH range of 7.07–9.6. Notably, the peak current obtained at pH 3.01 was higher than that at pH 7.07–9.6, confirming that higher peak currents were recorded at low pH values (pH 3.01 and 5.23) than the high pH (pH 7.07–9.6). This outcome may be due to the electrostatic interaction between melamine and the protonated ACE at low pH values, and the repulsion between the deprotonated ACE and melamine at high pH. More importantly, the highest peak current was obtained at pH 5.23 and subsequent electroanalysis was conducted at this pH (Fig. 3b). Meanwhile, a linear relationship with a slope of −37.3 mV pH^−1^ was recorded between the peak potential (*E*_p_) and pH. Because this slope is closer to 30 mV pH^−1^ than 59 mV pH^−1^, it can be inferred that 2 electrons per proton were involved in the rate-determining steps for the electrooxidation of ACE, as illustrated in Scheme 2.^27^ The participation of an unequal number of protons and electrons can be attributed to the more complex redox process than the common 2-electron/2-proton mechanism.

### 3.5 Impact of scan rate

**Fig. 4:**
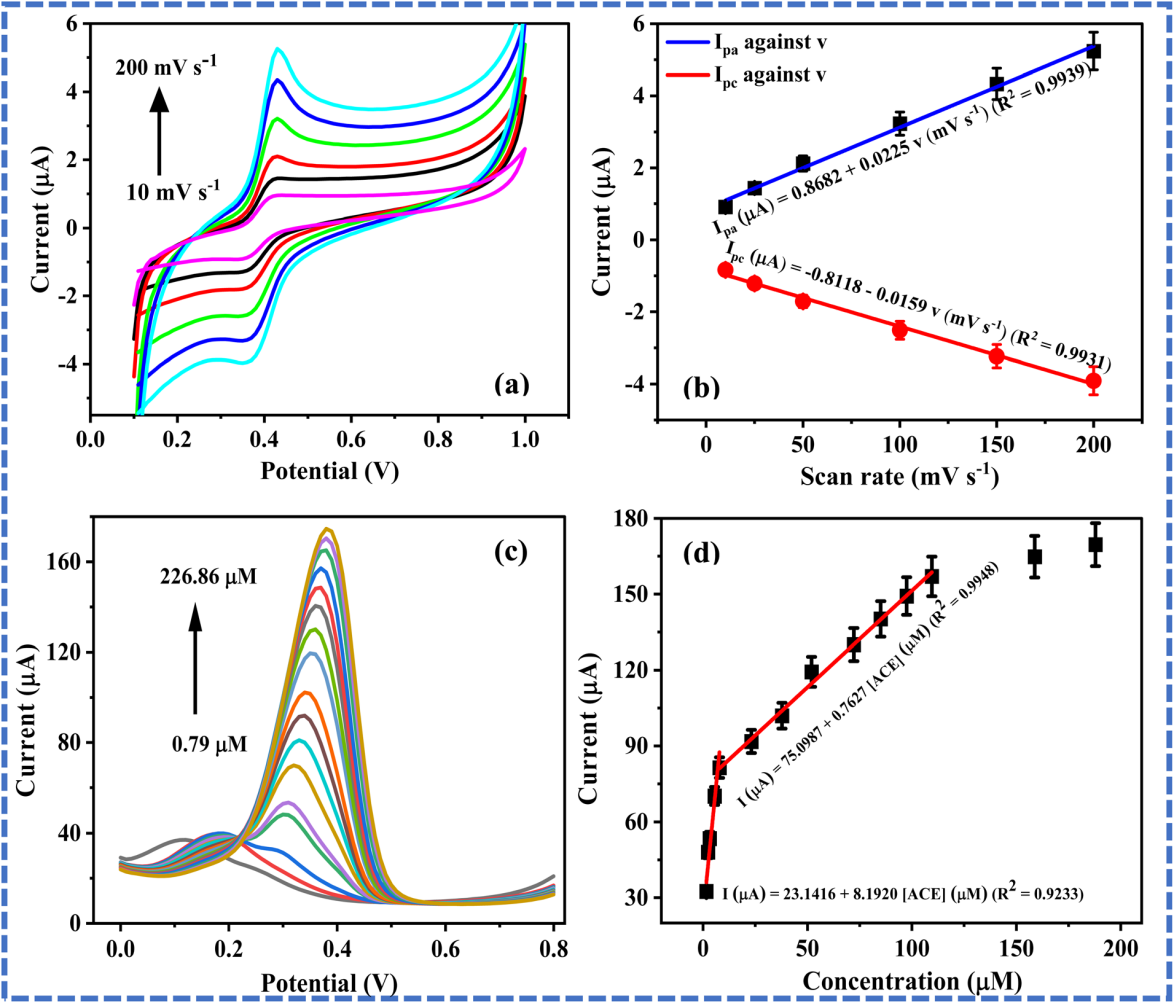
(a) Cyclic voltammograms of 75 µM ACE over a scan rate range of 25–450 mV s^−1^ (pH 5.23), (b) plot of peak current against scan rate, (c) differential pulse voltammograms of varying ACE concentrations (0.79–226.86 µM) at EPM/GCE, and (d) plot of ACE peak currents against concentration.

The impact of a variation in scan rate (*v*) on ACE peak current was investigated by recording the cyclic voltammograms of 75 µM ACE over a scan rate range of 10–200 mV s^−1^. As shown in Fig. 4a, the peak current increased with an increase in *v*. Similarly, the peak current of ACE increased with *v* (Fig. 4b) and the square root of *v* (*v*^1/2^) (Fig. S3a). The correlation coefficients of the plot of I_pa_ against *v* and that of I_pa_ against *v*^1/2^ were 0.9939 and 0.9912, respectively, confirming that the electroanalysis of ACE at EPM/GCE was a surface-confined process. Also, the plot of log *I*_pa_ against log *v* gave a slope of 0.59, as shown in Fig. S3b. This slope is close to the theoretical value for a diffusion-controlled process (0.5), suggesting that the electroanalysis of ACE at the proposed sensor is a surface-confined process. This outcome indicates that the oxidation of ACE at EPM/GCE is both surface-confined and diffusion-controlled.

### 3.6 Impact of concentration changes

The influence of concentration changes on the peak current of ACE at EPM/GCE was investigated by conducting differential pulse voltammetry over a concentration range of 0.79–226.86 µM, as shown in Fig. 4c. Notably, the peak current of ACE increased with an increase in the concentration of ACE ([ACE]). As illustrated in Fig. 4d, the peak current increased with an increase in [ACE] over the linear dynamic range (LDR) of 1.58–7.86 µM and 15.57–158.8 µM. The emergence of two LDRs is an indication that the mechanism of ACE oxidation at the EPM/GCE changes with an increase in [ACE]. The limit of detection (LOD) of ACE at the proposed sensor was estimated from the calibration plot of the lower LDR using the mathematical expression 3*δ*/*m*, where *δ* and *m* depict the standard error of the intercept and the slope of the calibration curve, respectively. The LOD of ACE recorded at EPM/GCE over this LDR was estimated as 1.46 µM Table 2. This LOD is comparable to that of some ACE sensors in the literature (Table 1). Noteworthy, the micromolar LOD of the proposed sensor indicates its suitability for the micromolar determination of ACE.

**Table 2:** Comparison of the performance of the proposed sensor with those of the recently reported ACE sensors^a^

Electrode	Method	LOD (µM)	LDR (µM)	References
Asp-MWCCNTs/IL/ITO/GCE	CV	0.019	0.132–1.98	3
GCE/Zn-MOF	DPV	0.1	1–50	28
MIP/rGO/GCE	DPV	0.01	0.03–0.2	6
SCB-activated biochar/GCE	CA	2.5	5–950	29
GR-PVP/ABPE	SDLSV	0.12	5–700	1
LSGE	SWV	0.044	0.1–10	2
α-Fe_2_O_3_/CHT/PANI/ITO	PM	5.7	5–100	30
EPM/GCE	DPV	1.46	1.58–7.86; 15.57–158.8	This work

a LSGE-laser-scribing technology electrode; GR-graphite; PVP-polyvinylpyrrolidone; ABPE-acetylene black paste electrode; SCB-sugarcane bagasse; CA-chronoamperometry; SWV-square wave voltammetry; SDLSV-second derivative linear sweep voltammetry; MIP-molecularly imprinted polymer; rGO-reduced graphene oxide; MOF-metal–organic framework; Asp-asparagine; MWCNTs-multi-walled carbon nanotubes; PM-potentiometric; IL-ionic liquid; ITO-indium tin oxide substrate; and CHT-chitosan.

### 3.7 Real sample analysis

**Fig. 5:**
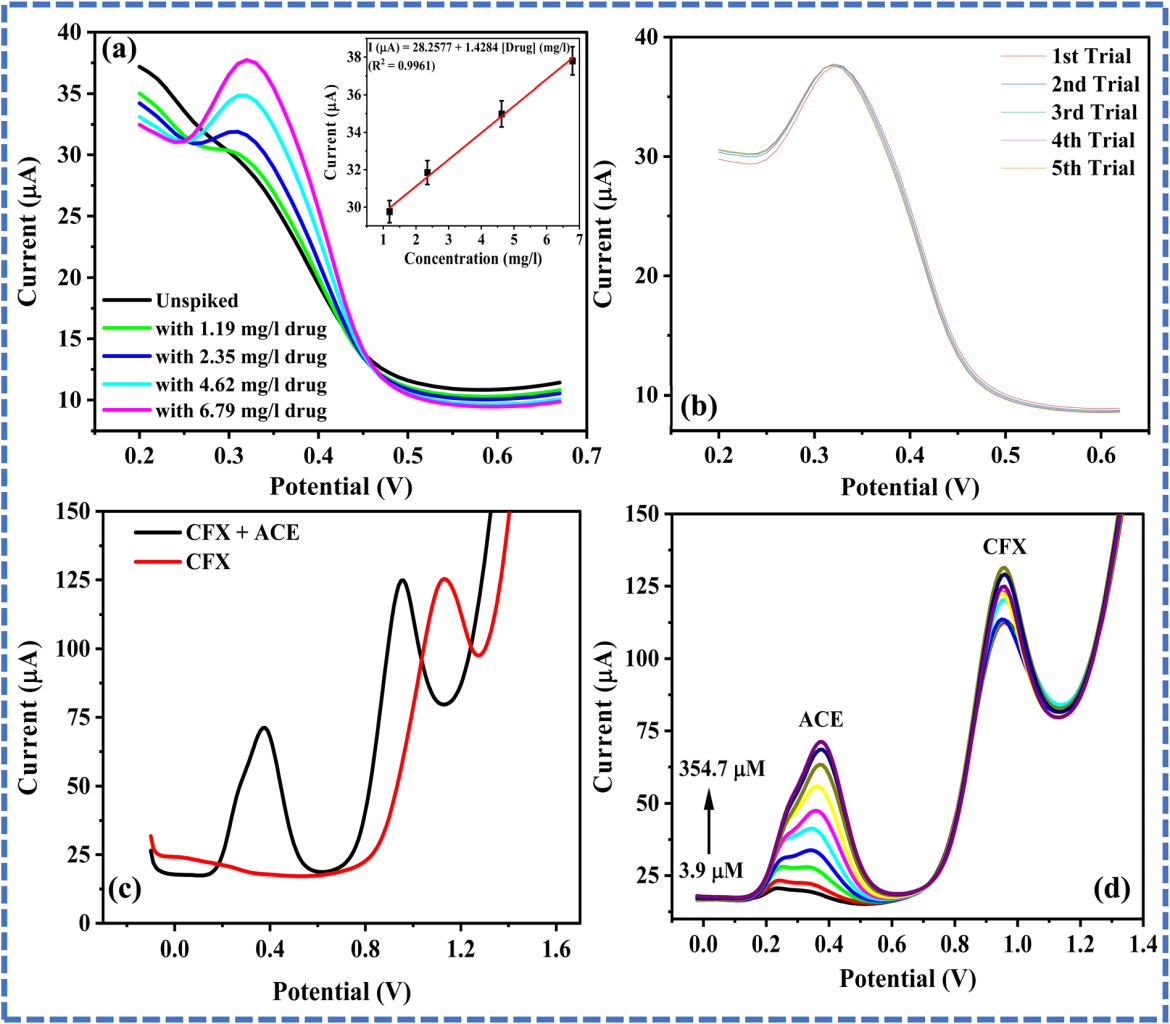
Differential pulse voltammogram of (a) unspiked tap water and tap water spiked with 1.19–6.79 mg L^−1^ drug (inset: plot of peak current against concentration of drug), (b) 6.8 mg L^−1^ drug (recorded five times), (c) 1 mM CFX and a mixture of 350 µM ACE and 1 mM CFX at EPM/GCE (pH 5.23), and (d) varying ACE concentrations (3.9–354.7 µM) in the presence of 1 mM CFX.

Real sample analysis of ACE was conducted with the drug sample, tap water spiked with the drug and waste water spiked with the drug sample. Electroanalysis of ACE in tap water sample spiked with known concentrations of a renowned paracetamol brand produced by Adcock Ingram (South Africa) was conducted using DPV. As shown in Fig. 5a, the peak current increased with an increase in the concentration of the drug sample from 1.19–6.79 mg L^−1^, suggesting that the electroanalysis of ACE in water samples at EPM/GCE is feasible. Also, the analysis of 6.8 mg L^−1^ drug sample was done using DPV, and the percentage relative standard deviation (% RSD) of the peak currents of ACE in the drug sample was 0.2%, confirming the repeatability of the current signal at the EPM/GCE. Analysis of ACE in a wastewater sample was also conducted by spiking the wastewater sample with 5.5 µM ACE and analyzing the spiked sample with DPV over five trials (Fig. 5b). Also, ACE electroanalysis in wastewater was conducted. The percentage ACE recovery from the wastewater sample was 92% with a % RSD of 0.5%. This percentage recovery agrees with that of the ACE recovery at modified electrodes in the literature.^3,31,32^

### 3.8 ACE electroanalysis in the presence of CFX

CFX is an antimicrobial formulation found in the environment at a considerably high concentration due to its low susceptibility to degradation by bacteria. Consequently, CFX is considered a potential interferon to ACE in water samples.^33^ Simultaneous electroanalysis of ACE and CFX was done with DPV to investigate the feasibility of applying EPM/GCE to ACE and CFX detection. Initially, individual CFX detection was done and the peak current was recorded at 1.13 V. In a mixture of ACE and CFX, ACE and CFX signals emerged at 0.37 and 0.95 V, respectively (Fig. 5c). The variation in the peak potentials at which the CFX peak current was recorded individually and simultaneously may be due to the competition between ACE and CFX for the electroactive surface of the EPM/GCE during electrooxidation. This competition is due to the π–π interaction between the analytes and the modified electrode. It is also worth noting that the CFX peaks were sharper than the ACE peak, and that there was a little difference in the peak current of CFX in the absence and the presence of ACE. Additionally, the ACE peak currents increased with an increase in [ACE], as shown in Fig. 5d.

**Fig. 6:**
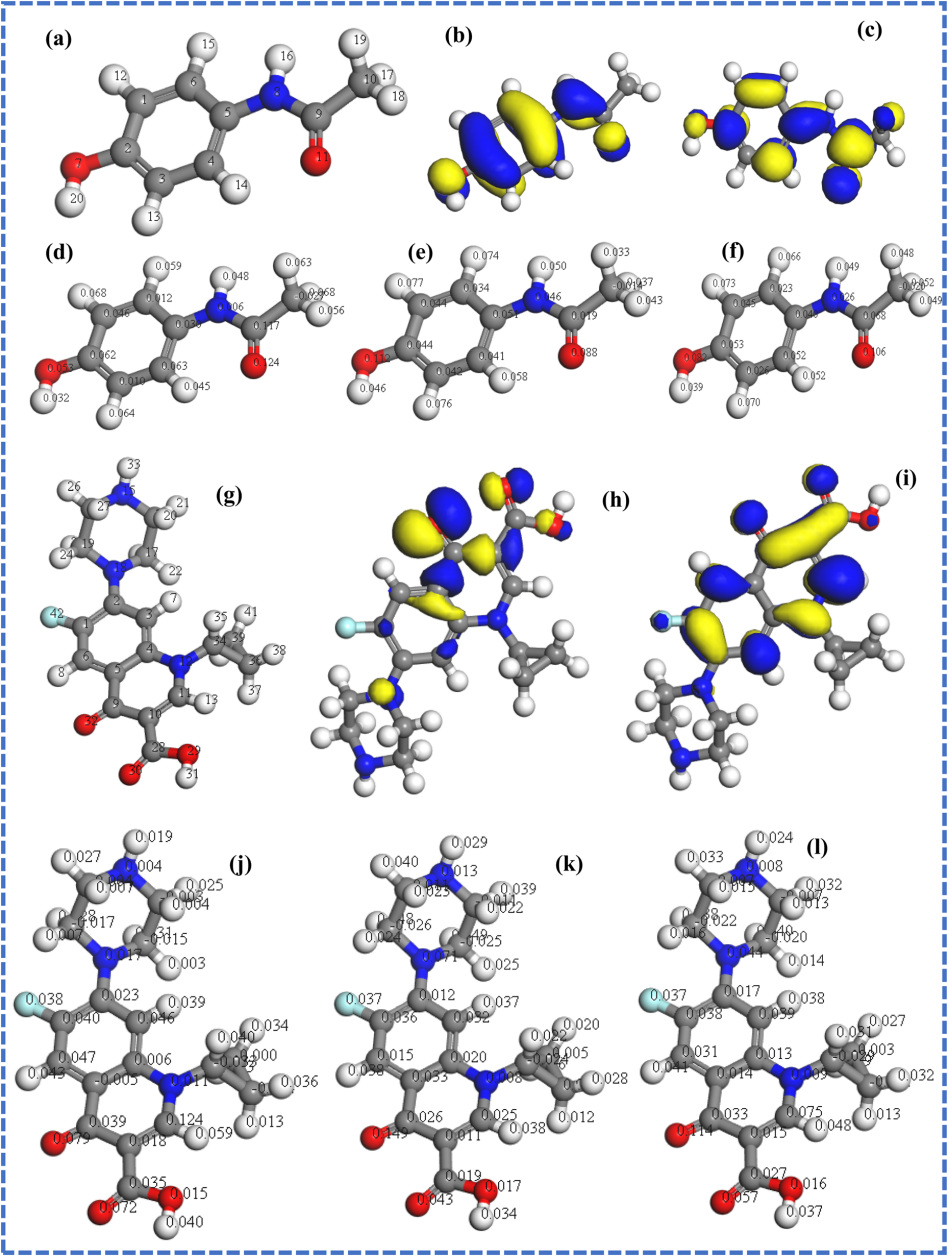
(a) Optimized structure, (b and c) frontier molecular orbitals (HOMO and LUMO) of ACE, and optimized structure of ACE showing Fukui indices: (d) *f*_k_^+^, (e) *f*_k_^−^ and (f) *f*^0^_k_. (g) Optimized structure (h and i) frontier molecular orbitals (HOMO and LUMO) of CFX, and optimized structure of CFX showing Fukui indices (j) *f*_k_^+^, (k) *f*_k_^−^ and (l) *f*^0^_k_.

**Fig. 7:**
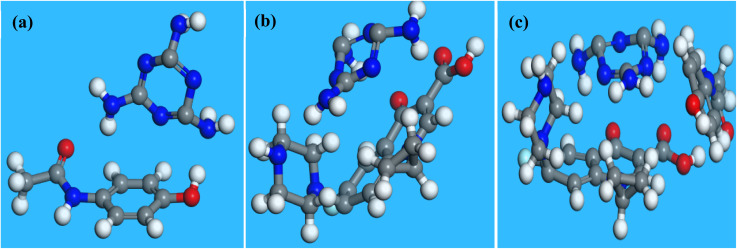
Structure of the melamine–ACE, melamine–CFX and melamine–CFX–ACE complexes after adsorption simulation.

To investigate the reason behind the persistent affinity of the proposed sensor for CFX in the presence of ACE, adsorption of the analytes individually and simultaneously was simulated. Fig. 6a-l shows the optimized structure, frontier molecular orbitals (HOMO and LUMO) and Fukui indices of CFX and ACE. The HOMO–LUMO gaps (Δ*E*) of CFX and ACE are 2.761 and 3.818, respectively, suggesting that CFX is more reactive than ACE (Table 3). The sites identified for nucleophilic attack on ACE are C2, C4, C9, H12, H17, and H19, while the preferred sites for electrophilic attack are H12, H13, H15, O7 and O11 (Fig. 6d and e), respectively. The sites for radical attack on ACE are C9, H12, H13, H15, O7 and O11 (Fig. 6f). In CFX, the sites identified for nucleophilic attack are C1, C3, O30, O32, H31, H13 and H35, while the active regions for electrophilic attack are O30, O32, N18, H26, H25 and H23 (Fig. 6j and k), respectively. The sites for radical attack on ACE are O30, O32, H13, H8, C11, N18 and H23 (Fig. 6l). The comparison of the Fukui indices of CFX and ACE indicate that O30 and O32 in CFX are better sites for nucleophilic attack than all the atoms in ACE. Similarly, O30 on CFX is a more preferable site for electrophilic and radical attack than any site in ACE, confirming the superiority of the reactivity of CFX over ACE. These outcomes also indicate that CFX may be preferably electrooxidized at the modified electrode over ACE, as evident by the intensity of CFX relative to that of ACE. The simulated ACE–melamine, CFX–melamine and ACE–CFX-melamine complexes are illustrated in Fig. 7. As suggested by the structure of the complexes in Fig. 7a and b, the hydrogen bonding between the nitrogen atoms in melamine and the hydroxyl groups in the analytes enabled the individual adsorption of ACE and CFX on melamine. The alignment of CFX with melamine also showed that the adsorption of CFX on melamine may be influenced by the π–π interaction between CFX and melamine. Meanwhile, the adsorption of CFX was more favoured in a mixture of CFX and ACE, as shown by the position of CFX relative to that of melamine in the ACE–CFX–melamine complex (Fig. 7c). Additionally, the adsorption energy and total energy of the CFX–melamine complex were lower (more negative) than that of the ACE–melamine complex, confirming the affinity of the proposed sensor for CFX (Table 3). Understandably, the adsorption energy of melamine–CFX–ACE was lower than that of melamine–ACE and melamine–CFX due to the abundance of active.

**Table 3:** Frontier molecular orbital energies of analytes and the adsorption energies of complexes^a^

Structure	*E*_HOMO_ (eV)	*E*_LUMO_ (eV)	Energy gap, Δ*E*	Adsorption energy (kJ mol^−1^)	Total energy (kJ mol^−1^)
ACE	−4.682	−0.864	3.818	—	—
CFX	−4.637	−1.876	2.761	—	—
Melamine–ACE	—	—	—	−22.392	−62.908
Melamine–CFX	—	—	—	−32.536	−122.764
Melamine–CFX–ACE	—	—	—	−62.846	−193.589

a Sites on ACE and CFX for adsorption on melamine. Despite the affinity of the proposed sensor for CFX, ACE electroanalysis was conducted in the presence of CFX, albeit with some interference with the intensity of the ACE signal. As shown in Fig. 5d, the peak current of ACE at EPM/GCE increased with an increase in [ACE] over a concentration range of 3.9–354.7 µM, while a slight increase in CFX current response was observed. These outcomes confirmed that ACE determination in the presence of CFX is feasible at EPM/GCE.

### 3.9 Repeatability, reproducibility and stability studies

**Fig. 8:**
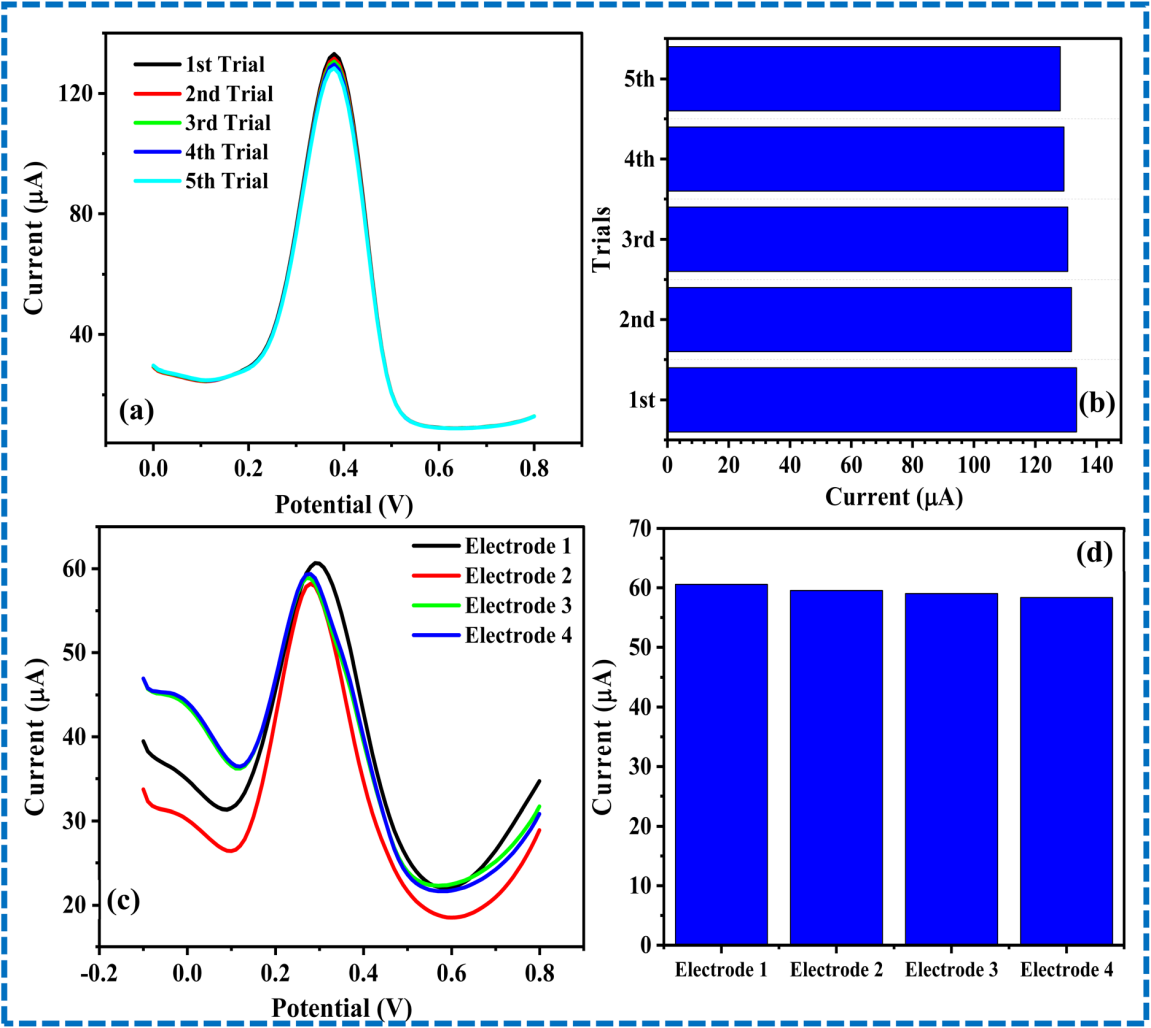
(a) Differential pulse voltammograms of 85 µM ACE over EPM/GCE recorded five times, and (b) chart showing the peak currents recorded in (a). (c) Differential pulse voltammograms of 10 µM ACE over four different EPM/GCE, and (d) chart showing the peak currents recorded in (c).

The repeatability of the EPM/GCE was investigated by recording the DPV of ACE at the proposed sensor repeatedly over five trials, as illustrated in Fig. 8a. The peak currents of ACE recorded after the five trials were very close, as illustrated in Fig. 8b. The % RSD of the current signals was estimated to be 1.37%, suggesting that the current obtained at EPM/GCE is repeatable with negligible error margin. Notably, the % RSD recorded in the current sensor is similar to that of the ACE sensors in the literature.^17,34,35^

The reproducibility of EPM/GCE was investigated by conducting DPV with four replicate sensors (Fig. 8c). As illustrated in Fig. 8d, there was little discrepancy in the current signal recorded at the respective modified electrodes. Specifically, the % RSD of these current signals was 1.79%. This % RSD is comparable to that of ACE sensors found in the literature.^36,37^

The stability of the current response obtained at the proposed sensor after 30 consecutive CV scans was recorded to establish the resistance of the proposed sensor to fouling. As shown in Fig. S3c, the peak current recorded at the end of the 30th scan was about 92% of the peak current of the first cycle. This outcome indicates that the proposed sensor exhibited negligible current loss due to possible fouling by the ACE oxidation product. Also, this result suggests that a stable current signal can be obtained at EPM/GCE after continuous application of this sensor for ACE electroanalysis.

## 4. Conclusion

Electrochemical deposition of PM on a bare GCE *via* an adsorption-assisted route was attempted in this study for the first time. Compared to CPM, the novel EPM offered superior electrocatalytic activity toward ACE electrooxidation. The LOD of ACE at the EPM/GCE was 1.46 µM, confirming its suitability for the micromolar determination of ACE. The electroanalysis of ACE in a renowned drug sample confirmed the suitability of the EPM/GCE for ACE quality control after its production. Also, the proposed sensor gave a satisfactory percentage ACE recovery (92%) in a wastewater sample. EPM/GCE offered high reproducibility in ACE and drug sample, remarkable repeatability in ACE and excellent stability after several CV cycles, confirming its reliability for ACE determination in real-life samples. The overall performance of the proposed sensor in ACE electroanalysis and its capability for simultaneous ACE and CFX determination position it as a potential tool for quality control in the production of pharmaceutical formulations. Theoretical studies confirmed the suitability of melamine for GCE modification, and the preference of EPM/GCE for CFX determination, necessitating the application of the proposed sensor for CFX determination in future studies.

## Conflicts of interest

There are no conflicts to declare.

## Supplementary Material

RA-015-D5RA07827J-s001

## Data Availability

Data will be made available upon reasonable request. Supplementary information (SI): experimental data including anodization *i*–*t* curve, voltammograms for the optimization of electropolymerization parameters, scan rate studies plots and stability sudies voltammograms. See DOI: https://doi.org/10.1039/d5ra07827j.
