# Supplementary material for: Pioneering adsorption-assisted electrochemical polymerization approach for the fabrication of an acetaminophen sensor: experimental and theoretical studies
Source: RSC Adv. 2025 Dec 11;15(57):49441–54. doi: 10.1039/d5ra07827j (PMC12696567; doi:10.1039/d5ra07827j)
Supplement: RA-015-D5RA07827J-s001 [file RA-015-D5RA07827J-s001.pdf]

Supplementary Information (SI) for RSC Advances.
This journal is © The Royal Society of Chemistry 2025

Supplementary Information

A pioneer adsorption-assisted electrochemical polymerization approach to the fabrication of acetaminophen sensor: Experimental and theoretical studies

Saheed E. Elugoke[1*], Usisipho Feleni[2], Abolanle S. Adekunle[2,3], Thabo T.I. Nkambule[2], Bhekie B. Mamba[2] and Eno E. Ebenso[1,*]

[1]Centre for Materials Science, College of Science, Engineering and Technology (CSET), Johannesburg 1710, South Africa

[2]Institute for Nanotechnology and Water Sustainability, College of Science, Engineering and Technology (CSET), Johannesburg 1710, South Africa

[3]Institute for Catalysis and Energy Solutions (ICES), College of Science, Engineering and Technology (CSET), Johannesburg 1710, South Africa

* Corresponding author: Centre for Material Science, College of Science, Engineering and Technology, University of South Africa, Johannesburg 1709, South Africa. Email: S.E. Elugoke-elugokesaheed@gmail.com, elugose@unisa.ac.za; Prof. E.E. Ebenso- ebensee@unisa.ac.za

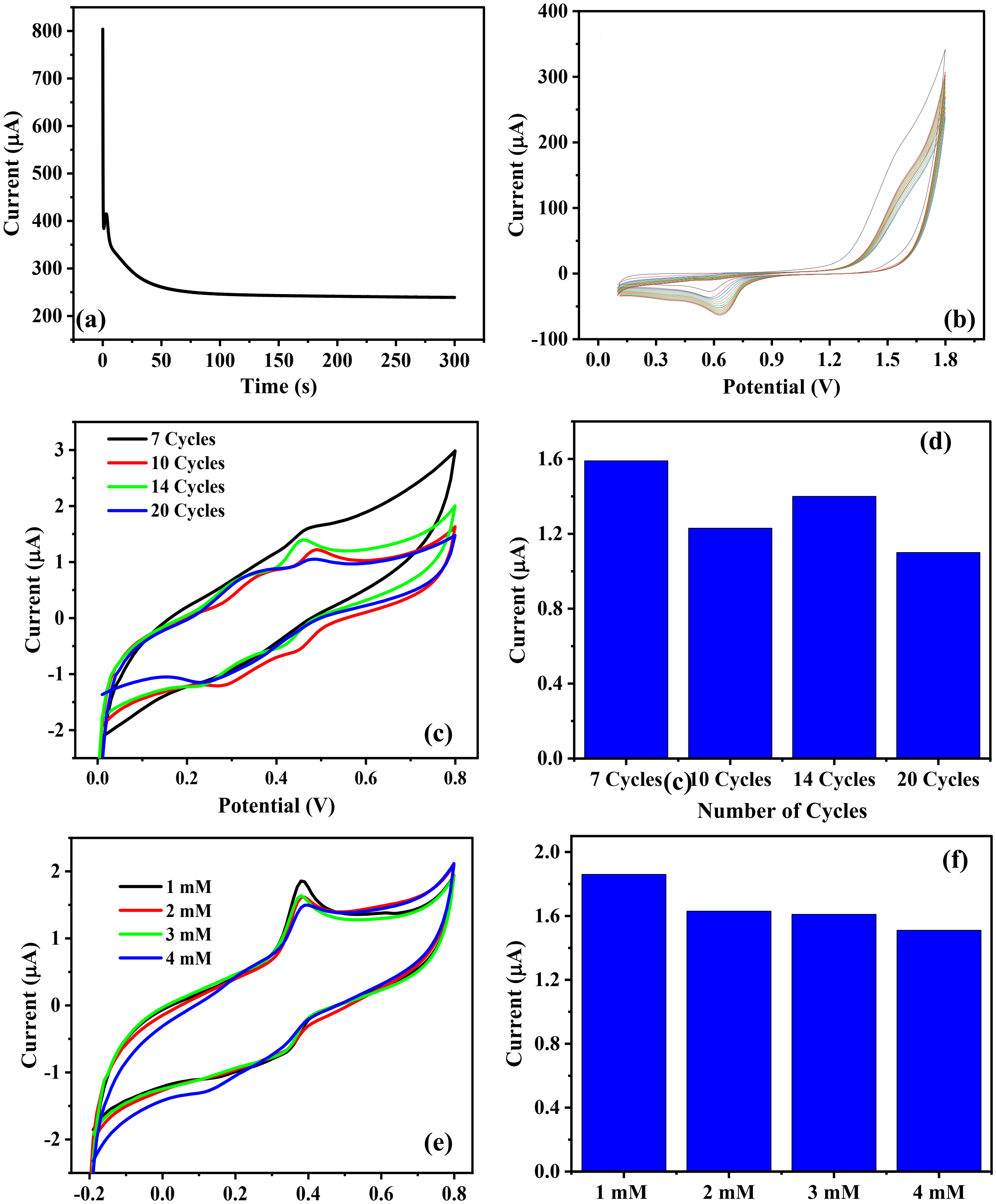

(a)
Current (µA)
Time (s)
800
700
600
500
400
300
200
0
50
100
150
200
250
300
(b)
Current (µA)
Potential (V)
400
300
200
100
0
-100
0.0
0.3
0.6
0.9
1.2
1.5
1.8
(c)
7 Cycles
10 Cycles
14 Cycles
20 Cycles
Current (µA)
Potential (V)
3
2
1
0
-1
-2
0.0
0.2
0.4
0.6
0.8
(d)
Current (µA)
Number of Cycles
1.6
1.2
0.8
0.4
0.0
7 Cycles
(c)
10 Cycles
14 Cycles
20 Cycles
(e)
1 mM
2 mM
3 mM
4 mM
Current (µA)
Potential (V)
2
1
0
-1
-2
-0.2
0.0
0.2
0.4
0.6
0.8
(f)
Current (µA)
Melamine concentration
2.0
1.6
1.2
0.8
0.4
0.0
1 mM
2 mM
3 mM
4 mM

Figure S1: (a) I-t curve for GCE anodization. (b) Cyclic voltammograms (14 CV cycles) of EPM/GCE fabricated after electropolymerization at a scan rate of 100 mV s^{-1}. Cyclic voltammograms of 7.62 μM ACE at EPM/GCE using varying (c, e) CV cycles and melamine concentration at 25 mV s^{-1}. Plots of currents against (d, f) number of CV cycles and melamine concentration

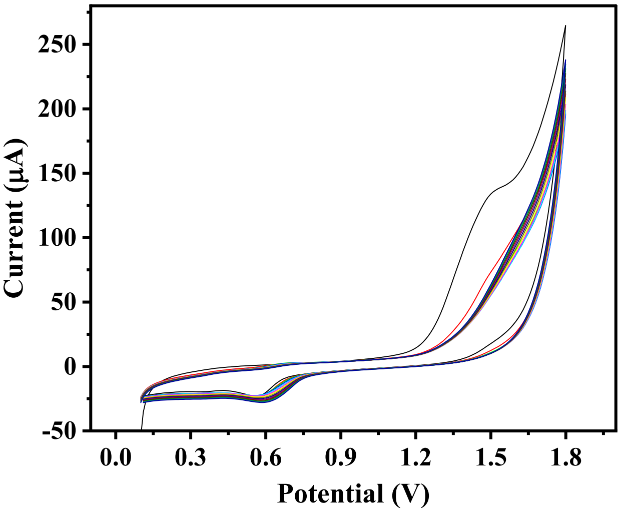

Figure S2: I-t curve for GCE oxdization. Cyclic voltammograms (14 CV cycles) of CPM/GCE after electrodeposition at a scan rate of 100 mV s-1

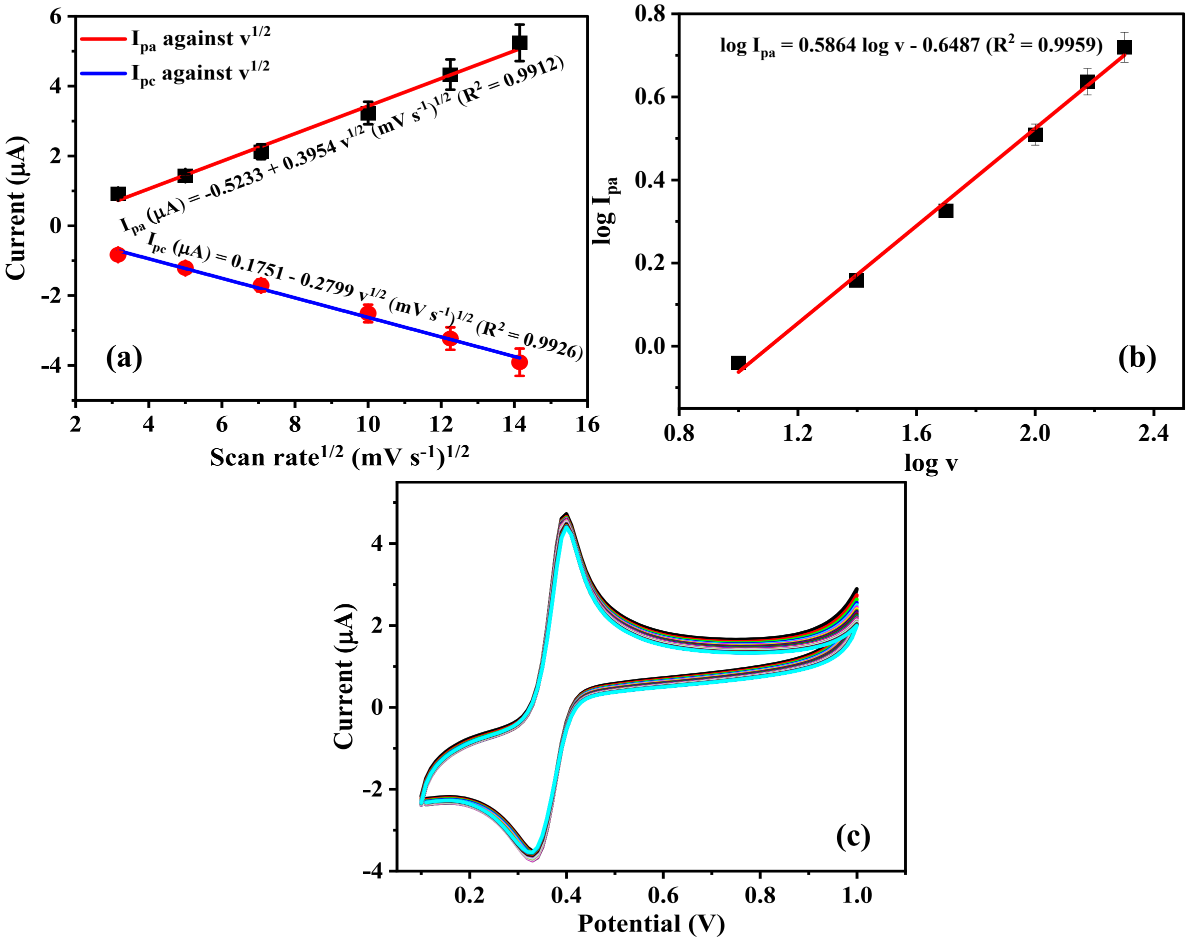

Figure S3: Plot of (a) peak currents against square root of scan rate and (b) log I_{pa} against log v. (c) Cyclic voltammograms (30 CV scans) of 0.16 mM ACE at EPM/GCE
